# The Pilot Survey of the Perception on the Practice Pattern, Diagnosis, and Treatment on Korean Medicine Insomnia: Focusing on the Difference between Korean Medical Neuropsychiatry Specialists and Korean Medical General Practitioners

**DOI:** 10.1155/2018/9152705

**Published:** 2018-02-11

**Authors:** Jung-Hwa Lim, Jin-Hyung Jeong, Sang-Ho Kim, Kyeong-Ok Kim, Seung-Yeon Lee, Sang-Hyup Lee, Bo-Kyung Kim

**Affiliations:** ^1^Department of Neuropsychiatry, School of Korean Medicine, Pusan National University, Yangsan, Republic of Korea; ^2^Department of Oriental Neuropsychiatry, College of Oriental Medicine, Dong-Eui University, Busan, Republic of Korea; ^3^Department of Oriental Neuropsychiatry, College of Korean Medicine, Daegu Haany University, Gyeongsan, Republic of Korea; ^4^Department of Oriental Neuropsychiatry, College of Oriental Medicine, Dong-shin University, Naju, Republic of Korea; ^5^Department of Oriental Pediatrics, College of Oriental Medicine, Dong-Eui University, Busan, Republic of Korea; ^6^Department of Korean Medical Classics, College of Oriental Medicine, Dong-Eui University, Busan, Republic of Korea

## Abstract

**Introduction:**

This study aims to investigate the clinical practice states on the diagnosis and treatment for insomnia between Korean medical general practitioners (KMGPs) and Korean medical neuropsychiatry specialists (KMNPSs).

**Methods:**

We distributed questionnaires via email or in person to 1,017 KMGPs and via email to 165 KMNPSs. We collected and analyzed responses from 305 (30.00%) KMGPs and 53 (32.12%) KMNPSs.

**Results:**

Most KMGPs and KMNPSs responded that the number of new patients visiting the clinic for treatment of insomnia was less than 10 per month (78.2%). Frequently utilized therapies for insomnia are acupuncture and herbal decoctions. Particularly acupoint GV20 and Guipi decoction were chosen with the highest response rate. There was no difference between KMNPSs and KMGPs in the traditional Korean medical diagnosis methods. However, KMNPSs utilized more various methods to diagnose, treat, and evaluate insomnia and educated more actively sleep hygiene compared to KMGPs.

**Conclusions:**

This survey showed how insomnia is currently diagnosed and treated in Korean medical care settings. Moreover, we identified some differences between KMNPSs and KMGPs. Further research is required to explore the underlying reasons for these discrepancies among KMDs and to improve the quality of Korean medical clinical practice in treating insomnia.

## 1. Introduction

Insomnia is one of the most prevalent neuropsychiatric disorders [1] and has many negative health, economic, and social consequences [2]. Epidemiological studies suggest that about 30% of the global population report general insomnia symptoms and that 10% of them report insomnia symptoms with daytime consequences [3]. In other epidemiological studies conducted in Japan and Korea, 13% and 23% of adults, respectively, are reported to suffer from insomnia [4, 5]. In a longitudinal study, approximately 40% of insomnia patients have reported suffering from chronic forms over time, and in three-year follow-up study, 46% patients reported persistent insomnia [3, 6].

Insomnia in itself causes impaired quality of life, increased risk of motor vehicle accident, and reduced job performance [7]. Furthermore, there is an established bidirectional relationship between insomnia and psychiatric disorders, particularly depression and anxiety; insomnia patients have the risk of developing depression as compared with their noninsomniac counterparts [8]. Insomnia is also associated with other physical comorbidities, such as myocardial infarction, hypertension, dyslipidemia, and diabetes [9].

Though pharmacotherapy and behavioral treatments can be effective for insomnia, they have limitations, including poor adherence, abuse, dependence, side effects, and difficulties inherent in their implementation [10, 11].

Because of these limitations of conventional treatments, many patients with insomnia have been using complementary and alternative medicine (CAM) remedies [12]. Insomnia is one of the top five medical conditions for which CAM is most frequently used. According to an analysis study of the 2007 National Health Interview Survey conducted in the United States, about 45% of adults who suffered from insomnia symptoms were estimated to receive CAM therapies per year [13].

Korean traditional medicine (KM) currently has a prominent place in the health care system of South Korea. Through the Medical Service Act (1951), KM is legitimately recognized as conventional medicine in the health care system. The independent six-year KM college education and four-year intern/resident KM hospital training program was established, corresponding to that of Western medicine; also, specialist qualifications in eight subfields—namely, internal medicine, gynecology, pediatrics, neuropsychiatry, acupuncture, ophthalmology-ears, nose, and throat- (ENT-) dermatology, rehabilitative medicine, and Sasang constitutional medicine—have been introduced since 2000 [14].

Up to 2016, 165 Korean traditional medical neuropsychiatry specialists (KMNPSs) have been educated. Furthermore, since 2008, Korean traditional medical clinical practice guidelines (CPGs) for some frequent diseases have been developed, supported by the Korean Ministry of Health & Welfare. Also, the project for developing additional Korean traditional medical CPGs has been underway since 2015 [15]. However, to date, Korean traditional medical CPGs, feasible for Korean traditional doctors (KMDs) to utilize in medical decision-making when treating patients with insomnia, have not been developed yet.

In order to ensure that clinicians adhere to the CPGs, the CPGs need to accurately reflect the clinical practice settings and requirement of clinicians. There have been no previous studies about how KMDs managed insomnia in their medical practices. Therefore, this research is aimed to explore how insomnia is currently diagnosed and treated in Korean medical care settings. This study would provide a foundation for the development of more acceptable Korean traditional medical CPGs for managing patients with insomnia.

## 2. Methods

### 2.1. Questionnaire

We carefully reviewed the available previous studies [16–19] regarding Korean traditional medical CPGs. The questionnaire was formulated by the Professors of Korean Medical Neuropsychiatry, who work in clinical practice as KMNPSs. The initially developed questionnaires were reviewed and edited by KMNPSs from Korean traditional medical hospitals, as well as by the scholarship director of the Pusan Association of Korean Medicine, on behalf of Korean traditional medical primary clinics. Prior to distributing the questionnaire, we received consultation from a panel comprised Korean medical general practitioners (KMGPs) and KMNPSs regarding the potential for understanding and response by questionnaire respondents. We also consulted an economic evaluation expert on the development of questionnaire items regarding economic evaluations.

### 2.2. Participants and Procedure

The KMGPs included in this study were registered members of the Pusan Association of Korean Medicine. We distributed the questionnaire to 1,017 KMGPs who were currently working in clinical practice via email and KM-related attendance in person. The respondents were rewarded for their participation with the beverage coupon worth 10,000 KRW.

We also sent the questionnaire via email to 165 KMNPSs, who were identified from the Korean Society of Oriental Neuropsychiatry. We distributed the questionnaire and collected responses from November 2, 2016 to November 18, 2016. In total, we collected responses from 305 (30.00%) KMGPs and 53 (32.12%) KMNPSs.

### 2.3. Statistical Analysis

All analysis was performed using SPSS for Windows version 23.00 (IBM Corp., Armonk, NY, USA). A *p* value of less than 0.05 was regarded as statistically significant. The data are presented as means ± SD or as the number of respondents (%). An analysis of variance test or unpaired Student's *t*-test was used for continuous variables, and a chi-square test was used for the categorical variables, with respect to possible differences in responses on the options for clinical practice between KMGPs and KMNPSs.

## 3. Results

### 3.1. The General Information about Korean Medical Status for Insomnia

The general characteristics of the 358 subjects are described in Table 1. Among the respondents, 265 (74%) were male and 93 (26%) were female; 163 were aged 40 to 49 years (45.5%); and 267 (74.6%) worked for a primary clinic; 153 (42.7%) were presented as having 10 to 20 years clinical career (Table 1).

In the questionnaire, we asked about the number of new patients who visited the clinic for insomnia in one month. The 280 (78.2%) participants replied that the number of patients who presented with insomnia as a chief complaint was less than 10 persons. Regarding the patients number with insomnia an accompanying symptom, 175 (49%) and 156 (43.7%) participants answered less than 10, and from 10 to 29, respectively.

We also asked KMDs about their first-time patients' confirmation status and the percentage of them who were taking sleeping pills. Among the respondents, 354 (99.2%) confirmed their patients' use of sleeping pills, while nine reported (2.5%) their patients did not take sleeping pills.

We questioned why insomnia patients would want to be treated by KM and allowed for duplicate responses. The KMDs answered that the patients who presented with insomnia wanted to improve their quality of sleep (63.3%), reduce their dosage of sleeping pills (61.3%), increase their sleeping time (51%), and alleviate related physical symptoms (49.9%). A dissatisfaction with sleeping pills (31.1%) was also a reason (Table 2).

We asked about the most frequently used code for the classification of Diagnosis in the National Health Insurance Database. As a result of our investigating, we found that G470 (disorders of initiating and maintaining sleep (insomnia)) and G479 (sleep disorder, unspecified) were the most preferred, and G478 (another sleep disorder), F510 (disorder of the sleep-wake schedule), F512 (nonorganic disorder of the sleep-wake schedule), F518 (other nonorganic sleep disorders), and F519 (nonorganic sleep disorder, unspecified) were also frequently used (Table 3).

We examined the prevalence of private insurance, including personal insurance and actual expense insurance, for insomnia patients. The majority (44.1%) of their patients did not have private insurance, while 23.5% of respondents said that less than 10% of their insomnia patients had private insurance.

### 3.2. How to Treat Insomnia according to KMGPs and KMNPSs: Diagnosis, Pattern Identification, Treatment, and Assessment

In our questionnaire, we also asked about the tools of insomnia diagnosis. The KMDs diagnosed insomnia mainly based on patients' symptoms (98.9%), and some used heart rate variability (HRV) (14.8%) or pulse diagnosis (12.6%) as diagnosis method. The KMNPSs used questionnaire (*p* < 0.001), the performance of an electroencephalogram (EEG) including neurofeedback (*p* = 0.023), and use of HRV (*p* < 0.001) with significantly higher rate compared to the KMGPs (Table 4).

Regarding the pattern identification of insomnia, KMDs most preferred the order of visceral pattern identification (57.5%), eight principles pattern identification (17.0%), and constitutional pattern identification (11.7%). There was no significant difference in pattern identification between KMGPs and KMNPs.

The KMDs reported using various treatment methods presented in Table 10. The use of acupuncture and herbal medicine decoctions were the options most frequently and importantly used by KMDs for insomnia; moxibustion, pharmacopuncture, dry cupping, wet cupping, uninsured herbal extract granules, and psychotherapy followed. The KMNPSs more actively used electroacupuncture (*p* < 0.001), auricular acupuncture (*p* = 0.048), Chuna (*p* = 0.019), Dao Yin exercise (*p* = 0.023), insured herbal extract granules (*p* < 0.001), uninsured herbal extract granules (*p* < 0.001), dry cupping (*p* = 0.005), meditation (*p* < 0.001), aromatherapy (*p* < 0.001), psychotherapy (*p* < 0.001), and biofeedback (*p* = 0.046) for treating insomnia as compared with the KMGPs (*p* < 0.05) (Table 5).

KMDs answered that the most frequently used treatment for insomnia was acupuncture (45.83%) closely followed by the use of herbal decoctions (22.48%). KMGPs used acupuncture for insomnia with higher rate compared to KMNPs (*p* = 0.005) (Table 6).

KMDs most frequently used general acupuncture and Sa-am acupuncture; O-Haeng acupuncture followed. KMNPSs made more significant use of general acupuncture as compared with KMGPs (*p* = 0.003). Also, the most frequently chosen acupoints by KMDs for treating insomnia were GV20 followed by PC60, HT07, EX-HN1, ST36, HT08, SP06, KI62, and BL62. KMNPSs significantly more often chose the HT07 (*p* = 0.001), KI06 (*p* = 0.003), and SP06 (*p* < 0.001) acupoints (Table 7).

KMDs selected Guipi decoction, Wendan decoction, Xiaoyao powder, Suanzaoren decoction, and the Chaihu Jia Longgu Muli decoction for the treatment of acute insomnia. Besides the above prescriptions, they also chose Tianwang Buxin pills, and Ganmaidazao decoction.

Guipi decoction was the prescription chosen most often by both KMGPs and KMNPSs, though KMNPs also used Tianwang Buxin pill (*p* < 0.001), Yokgansan (*p* < 0.001), Buxue Anshen decoction (*p* = 0.001), Ganmaidazao decoction (*p* = 0.026), Chaihu Jia Longgu Muli decoction (*p* < 0.001), and the Hwanglianjiedu decoction (*p* = 0.010) more than KMGPs (*p* < 0.05) (Table 8).

KMDs chose the Guipi decoction, Wendan decoction, Qingxin Lianzi decoction, Suanzaoren decoction, Xiaoyao powder, and Si-wu Anshen decoction to treat chronic insomnia. KMPNSs significantly more often chose the Gupi decoction (*p* = 0.015), Buxue Anshen decoction (*p* = 0.001), Chaihu Jia Longgu Muli decoction (*p* = 0.002), and Er ShenJiao Ji Dan (*p* = 0.016) as compared with KMGPs (*p* < 0.05) (Table 9).

Additionally, 98.3% of KMDs replied that they assess the treatment results based on the patient's statement. In their determination of treatment effect, they also considered the changing dosage of sleeping pills, changes in pulse diagnosis, and changes in face color. KMNPSs reported a higher utilization rate of questionnaires (*p* < 0.001), changes in sleeping pill dose (*p* < 0.001), and HRV (*p* < 0.001) as compared with KMGPs (*p* < 0.05) (Table 10).

### 3.3. What KMDs Think about the Management of Insomnia

KMDs most often replied that the time to response to treatment was from one to two weeks, or from two to three weeks; from three to four weeks followed. Regarding the time to take to respond to treatment, KMGPs most often replied from one to two weeks (30.0%), and the most frequent answer by KMNPSs was from three to four weeks (28.3%). There were no significant differences between KMGPs and KMNPSs in this respect.

KMDs most often replied that the minimal period to treat insomnia was from four weeks to two months, or from two to three months; one to four weeks followed. Regarding the minimal period to treat insomnia, KMGPs most often replied from two to three months (28.9%), and the most frequent answer of KMNPSs was from four weeks to two months (43.4%). There were no significant differences between KMGPs and KMNPSs in this respect.

KMDs replied that the most challenging aspect of treating insomnia was dealing with the cost burden to patients, delays in treatment effectiveness, and difficulties in objective evaluation regarding the treatment effect. KMPNSs showed a higher response rate regarding the delayed effectiveness as compared with KMGPs (*p* = 0.005) (Table 11).

KMDs thought that the most important factor for good prognosis in treating insomnia is the continuous intake of herbal medicine; also external environment change, continuous acupuncture and moxibustion, and rapport followed.

In conducting sleeping hygiene education and counseling, 49.6% of the total KMDs replied that they conduct it, and among them, KMNPSs (90.6%) conducted sleep hygiene education and counseling more frequently compared to KMGPs (42.4%) (*p* < 0.001) (Table 12).

KMDs replied that the appropriate duration for sleep hygiene education and counseling for insomnia is 18.02 ± 12.84 minutes/session and 1.43 ± 1.20 times/week, and the cost should be 16126.69 ± 14979.97 KRW/session. Regarding the appropriate duration, KMNPSs gave significantly shorter periods (7.26 ± 3.54 week) compared to KMGPs (8.54 ± 4.37 week) (*p* = 0.047) (Table 13).

## 4. Discussion

This research has strength in that it is the first-ever study to investigate the use of KM clinical practice methods in insomnia treatment and to compare perception on diagnosis, treatment approaches, assessment, and management of insomnia among KMGPs and KMPNSs in South Korea. Also this study has significance in that it involved a considerable number of KMGPs and KMPNSs, almost one-third of the registered members of the Pusan Association of Korean Medicine (30.00%), and the entire group of KMNPSs (32.12%).

Regarding the number of new patients visiting the clinic for per month, a considerable number of respondents (78.2%) reported less than 10 patients. Additionally, patients who have insomnia as an accompanying symptom are more prevalent than those with insomnia as a chief complaint.

This result implies that insomnia patients have little understanding of the use of KM treatments for insomnia and also suggests that both patients and KMDs might underestimate sleep problems. Actually, Jeong et al. [20] reported that Korean patients with insomnia were eight times more likely to use Western medicine than Korean Medicine, based on data collected in the Health Insurance Review and Assessment Service database from 2011 to 2013. Other previous reports [21] have suggested insomnia is typically underrecognized and underdiagnosed by health care professionals. Similarly, Morin et al. [22]. reported that a large number of insomnia patients tend not to seek medical help, or they delay treatment. A 2014 Australian survey reported that only 30% of insomnia patients had consulted a health professional [23].

Most of the KMDs (99.2%) involved in this study responded that they checked upon presentation whether insomnia patients had taken sleeping pills or not, and that a considerable number of the insomnia patients seeking KM treatment were using sleeping pills. The results showed that patients with insomnia who chose to use Korean medical treatment wanted to decrease their dosage of sleeping pills, besides the goal of improving sleep quality. Several studies also suggested that many patients with insomnia were seeking complementary alternative medicine for sleep complaints, because abuse, dependence, and adverse effects are major concerns with respect to pharmacotherapy use for insomnia [11, 24, 25].

Most of the KMDs reported that they usually used G 470 (disorders of initiating and maintaining sleep (insomnia)), 478 (other sleep disorders), and 479 (sleep disorder, unspecified) codes of the International Classification of Disease, 10th revision, for reporting to the National Health Care Insurance. Most psychiatric disorders are not covered by private insurance in South Korea. Also, patients do not want to be diagnosed with F codes because of existing stigma and prejudice against psychiatric illnesses.

Most of the participating KMDs reported diagnosing insomnia based on patients' complaints, though KMNPSs presented a higher rate of using sleep questionnaires, EEG instruments, and HRV as diagnosis tools than did KMGPs. The most frequently used pattern identification method was visceral pattern identification, and there was no significant difference between KMGPs and KMPNSs.

The top two therapeutic methods for insomnia which KMDs most frequently used were acupuncture (97.5%) and herbal decoctions (86.3%), and also these two therapeutic methods made up the greatest portion of total treatments.

KMNPSs reported a higher utilization of electroacupuncture, auricular acupuncture, Chuna, Dao Yin exercise, insured herbal extract granules, uninsured herbal extract granules, cupping therapies, meditation, aromatherapy, psychotherapy, and biofeedback as compared with KMGPs.

Regarding the assessment of therapeutic effect, most KMDs assessed based on patients' statements; meanwhile, KMNPSs more frequently made use of sleep questionnaires and reported changes in sleeping pill dosage, and HRV as assessment tools of therapeutic effect.

Roughly half of KMDs reported that they conducted sleep hygiene education for insomnia patients; however, KMNPSs showed more use of sleep hygiene education for insomnia patients as compared with KMGPs.

KMPNSs have to complete the four-year intern and residency course in KM hospital training program to achieve KMNPS board. Because of this, we thought that they would have had more opportunities to learn about sleep diagnosis tools or to obtain information about the latest trends for insomnia treatment over the course of their training program, and these could have influenced the differences in terms of diagnosis, therapeutic approach methods, assessment tools, and patient education implementation observed between KMGPs and KMNPSs.

KMDs reported that their most frequently used acupuncture method was general acupuncture and that GV20, PC06, and HT07 were the most frequently selected acupoints for insomnia treatment.

KMDs response regarding the use of herbal decoctions for acute insomnia was similar to that of herbal decoctions for nonacute insomnia. KMDs reported that Guipi decoction and Wendan decoctin were most frequently chosen for acute and nonacute insomnia. KMNPSs prescribed more various herbal decoctions used for psychiatric disorders, such as Yokgansan, Ganmaidazao decoction, and Chaihu Jia Longgu Muli decoction. With regard to preference for medicinal herbs to treat insomnia, Seed of* Zizyphus jujuba* Mill, Root stock of Cyperus rotundus L., and Aril of* Euphoria longan *(Lour) Steud were the top three most reported by KMDs.

Similar to in traditional Chinese medicine, the Korean medical treatment principal underlying herbal prescription and acupoints selection is pattern identification [26]. All diagnosis and therapies in Korean Medicine are based on the differential diagnosis of KM pattern identifications. Therefore, KMDs included in this study might choose herbal decoctions according to pattern identification, not phase of disease. This result was similar to that from the systematic review of Chinese and English literatures on traditional Chines medicine pattern differentiation [27]. The above systematic review [27] reported that, among herbal descriptions, Gui Pi tang and Wen Dan Tang were most commonly used, but the selection of herbs and acupoints was not correlated with pattern identification. We assumed that KMDs choose their herbal prescriptions based on KM pattern identification; however, they instead typically choose the herbs that have been added to main herbal prescriptions and the acupoints depending on their own clinical experience and patients' symptoms.

More than half of KMDs reported that the response time to KM treatment for insomnia was from one to three weeks. A considerable number of KMDs responded that the minimal length of KM treatment for insomnia was from four weeks to three months.

KMDs replied that the cost burden on patients, delays in treatment effect, and difficulties in objective assessment were the biggest challenges facing KM treatment for insomnia.

KMDs considered the continuous intake of herbal medicine and the making of an external environment change the most important contributing factors to successful treatment of insomnia.

Regarding sleep hygiene education, KMDs replied that the appropriate session time, session number per week, total period, and cost per session were 18.02 ± 12.84 minutes/session, 1.43 ± 1.20 sessions/week, 8.33 ± 4.27 weeks, and 16126.69 ± 14979.97 KRW/session, respectively.

The needs of the main users of CPG would be reflected to develop CPG for insomnia and to determine the clinical questions based on our results. The majority of KMGPs used to diagnose and evaluate insomnia disorder based on patients' complaints; therefore the researches to develop a standard tool of pattern identifications for insomnia for KMGPs are needed. The researches for identifying the treatment period to reach the minimal clinically important difference (MCID) after herbal medicine or acupuncture treatment are also needed. Acupuncture and herbal medicines are most frequently chosen as treatment methods for insomnia, but the appropriate therapeutic periods have not decided yet. Furthermore, the studies to testify the efficacy and safety of various remedies used by KMNPSs more frequently such as pharmacopuncture, meditation, Chuna, and biofeedback are required.

Because there are differences in sociocultural background and health care system with respect to traditional medicine among the countries, there may be the discrepancies in perceptions of clinical practice on insomnia between traditional medical doctors in other Asian countries and KMDs. Although this study aimed to investigate and compare the difference of perspective of clinical practice on insomnia among KMDs, our results could provide a reference for researchers or practitioners regarding managing insomnia patients at clinical setting and developing clinical research questions. Further survey studies to compare the perceptions of clinical practice on insomnia among Asian countries such as China, Twain, Hong Kong, and Japan are needed. Additionally, the international standard on the pattern identifications and evaluation tools on insomnia for multinational studies should be developed via future researches.

## 5. Limitations

This study is limited in the following aspects: first, the study subjects, KMGPs, were confined to those who work in Pusan City; thus this study may not fully represent the KM clinical practice status among the KMGPs nationwide. Nevertheless, Pusan is the second most-populated city in Korea, and the response rate to this study was relatively high (30.00%). Similar survey study [28] was aimed to identify the current status of Korean medical practice on dementia and mild cognitive impairment; the response rates were 3.6% (185 of 5146) in KMGPs and 21.8% (36 of 165) in KMNPSs, respectively.

Therefore this study could have representativeness in the demographic characteristics compared to previous studies.

Second, items included in the survey questionnaire for this research could be used to confirm the overall opinions and differences in clinical practice status among KMDs, but could not explain the concrete reasons behind these results. Further qualitative researches to find out the reasons as to why the differences in clinical practice among KMDs exist, and more in-depth exploration of KMDs who specialize in insomnia treatment, are needed.

## Figures and Tables

**Table 1:** General demographic characteristics.

Category	Classification	*n* (358)	%
Age group (years)	30 under	22	6.1
	30–39	111	31.0
	40–49	163	45.5
	50–59	57	15.9
	60 or over	5	1.4

Gender	Male	265	74.0
	Female	93	26.0

Affiliation	KM primary clinic	267	74.6
	KM hospital	56	15.6
	University, laboratory	13	3.6
	Public health service, military service.	6	1.7
	Convalescent hospital	12	3.4
	Leave of absence of job	4	1.1

Clinical career (yrs.)	<5	44	12.3
	5~10	91	25.4
	10~20	153	42.7
	20~30	65	18.2
	30<	5	1.4

**Table 2:** Why the patients with insomnia want to be treated by the Korean Medicine.

Category	*n*	%
To increase sleeping time	182	51.0
To improve quality of sleep	226	63.3
To reduce dosage of sleeping pill	219	61.3
Unsatisfaction with sleeping pills	111	31.1
To alleviate related physical symptoms	178	49.9
Other	2	0.6

**Table 3:** Korean Classification of Diagnosis code for Korean National Insurance coverage.

KCD code	*n*	%
G470		
Disorders of initiating and maintaining sleep (insomnia)	229	64.0
F510		
Disorder of the sleep-wake schedule	13	3.6
G478		
Other sleep disorder	24	6.7
F518		
Other nonorganic sleep disorders	2	.6
G479		
Sleep disorder, unspecified	74	20.7
F519		
Nonorganic sleep disorder, unspecified	5	1.4
F 512		
Nonorganic disorder of the sleep-wake schedule	11	3.1

**Table 4:** The diagnosis methods for insomnia and the difference between KMGP and KMNPS.

Diagnostic tool	Total (*n* = 358)	KMGP (*n* = 305)	KMNPS (*n* = 53)	*χ* ^2^	*p*
Patient's chief complaints	354 (98.9%)	301 (98.7%)	53 (100.0%)	.703	.402
Questionnaire	23 (6.4%)	3 (1.0%)	20 (37.7%)	101.450	.000
EEG including neurofeedback	10 (2.8%)	6 (2.0%)	4 (7.5%)	5.178	.023
HRV	53 (14.8%)	29 (9.5%)	24 (45.3%)	45.818	.000
Refer to other department	1 (.3%)	1 (.3%)	0 (0.0%)	.174	.676
Pulse diagnosis	45 (12.6%)	39 (12.8%)	6 (11.3%)	.088	.766
Other	10 (2.8%)	8 (2.6%)	2 (3.8%)	.220	.639

KMGP: Korean medical general practitioner, KMNPS: Korean medical neuropsychiatry specialist, and HRV: heart rate variability.

**Table 5:** The treatment method for insomnia and the difference between KMGP and KMNPS.

Treatment method	Total (*n* = 358)	KMGP (*n* = 305)	KMNPS (*n* = 53)	*χ* ^2^	*p*
Acupuncture	349 (97.5%)	297 (97.4%)	52 (98.1%)	.100	.752
Electroacupuncture	57 (15.9%)	40 (13.1%)	17 (32.1%)	12.126	.000
Auricular acupuncture	50 (14.0%)	38 (12.5%)	12 (22.6%)	3.896	.048
Intradermal acupuncture	34 (9.5%)	31 (10.2%)	3 (5.7%)	1.065	.302
Warm acupuncture	8 (2.2%)	8 (2.6%)	0 (0.0%)	1.422	.233
Burning acupuncture	3 (0.8%)	2 (0.7%)	1 (1.9%)	.824	.364
Acupotomy	1 (0.3%)	1 (0.3%)	0 (0.0%)	.174	.676
Pharmacopuncture	109 (30.4%)	98 (32.1%)	11 (20.8%)	2.760	.097
Bee venom herbal acupuncture	7 (2.0%)	7 (2.3%)	0 (0.0%)	1.241	.265
Chuna	56 (15.6%)	42 (13.8%)	14 (26.4%)	5.471	.019
Dao Yin exercise	10 (2.8%)	6 (2.0%)	4 (7.5%)	5.178	.023
Insured herbal extract granules	74 (20.7%)	51 (16.7%)	23 (43.4%)	19.594	.000
Uninsured herbal extract granules	88 (24.6%)	63 (20.7%)	25 (47.2%)	17.122	.000
Herbal medicine decoction	309 (86.3%)	260 (85.2%)	49 (92.5%)	1.985	.159
Wet cupping	81 (22.6%)	64 (21.0%)	17 (32.1%)	3.173	.075
Dry cupping	97 (27.1%)	73 (23.9%)	24 (45.3%)	10.516	.005
Moxibustion	163 (45.5%)	136 (44.6%)	27 (50.9%)	.735	.391
Needle embedding therapy	4 (1.1%)	4 (1.3%)	0 (0.0%)	.703	.402
Ice & hot pack	27 (7.5%)	23 (7.5%)	4 (7.5%)	.000	.999
Meditation	35 (9.8%)	16 (5.2%)	19 (35.8%)	47.942	.000
TENS	24 (6.7%)	22 (7.2%)	2 (3.8%)	.854	.355
ICT	29 (8.1%)	25 (8.2%)	4 (7.5%)	.026	.873
Deep heat- diathermy	5 (1.4%)	5 (1.6%)	0 (0.0%)	.881	.348
Laser	4 (1.1%)	3 (1.0%)	1 (1.9%)	.333	.564
Aromatherapy	27 (7.5%)	11 (3.6%)	16 (30.2%)	45.756	.000
Psychotherapy	75 (20.9%)	48 (15.7%)	27 (50.9%)	33.794	.000
Biofeedback	4 (1.1%)	2 (0.7%)	2 (3.8%)	3.973	.046
Other	3 (0.8%)	2 (0.7%)	1 (1.9%)	.824	.364

KMGP: Korean medical general practitioner, KMNPS: Korean medical neuropsychiatry specialist.

**Table 6:** The Proportion of treatment method for insomnia and the difference between KMGP and KMNPS.

Treatment method	Total (*n* = 358)	KMGP (*n* = 305)	KMNPS (*n* = 53)	*t*	*p*
	Mean (SD) (%)	Mean (SD) (%)	Mean (SD) (%)		
Acupuncture	45.83 (23.90)	47.32 (23.97)	37.26 (21.81)	2.855	.005
Herbal decoction	31.44 (22.48)	31.16 (22.95)	33.06 (19.68)	−.545	.586
Insured herbal extract granules	13.11 (11.94)	13.12 (12.66)	13.06 (8.41)	.024	.981
Uninsured herbal extract granules	16.78 (15.87)	16.00 (15.75)	19.72 (16.23)	−1.125	.263
Moxibustion	12.85 (13.36)	12.35 (10.70)	15.31 (22.39)	−.767	.448
Cupping	11.58 (13.13)	11.87 (13.90)	10.46 (9.55)	.567	.571
Other	0.65 (3.55)	0.53 (2.95)	1.34 (5.89)	−.982	.330

KMGP: Korean medical general practitioner, KMNPS: Korean medical neuropsychiatry specialist.

**Table 7:** The acupuncture treatment methods, selected acupoints for insomnia, and the difference between KMGP and KMNPS.

Category	Classification	Total (*n* = 358)	KMGP (*n* = 305)	KMNPS (*n* = 53)	*χ* ^2^	*p*
Acupuncture treatment method	General acupuncture	264 (73.7%)	216 (70.8%)	48 (90.6%)	9.093	.003
	Auricular acupuncture	39 (10.9%)	30 (9.8%)	9 (17.0%)	2.375	.123
	Scalp acupuncture	32 (8.9%)	24 (7.9%)	8 (15.1%)	2.896	.089
	O-Haeng acupuncture	75 (20.9%)	68 (22.3%)	7 (13.2%)	2.252	.133
	Constitutional acupuncture	29 (8.1%)	28 (9.2%)	1 (1.9%)	3.243	.072
	Dong-Si acupuncture	36 (10.1%)	33 (10.8%)	3 (5.7%)	1.329	.249
	Sa-am acupuncture	122 (34.1%)	105 (34.4%)	17 (32.1%)	.111	.739
	Other	15 (4.2%)	15 (4.9%)	0 (0.0%)	2.721	.099

Acupuncture point	GV20	243 (67.9%)	202 (66.2%)	41 (77.4%)	2.565	.109
	Sishencong	121 (33.9%)	102 (33.6%)	19 (35.8%)	.106	.744
	HT07	158 (44.1%)	124 (40.7%)	34 (64.2%)	10.110	.001
	HT08	96 (26.8%)	81 (26.6%)	15 (28.3%)	.070	.791
	PC06	159 (44.4%)	131 (43.0%)	28 (52.8%)	1.911	.385
	ST36	102 (28.5%)	84 (27.5%)	18 (34.0%)	.914	.339
	BL62	77 (21.5%)	61 (20.0%)	16 (30.2%)	2.776	.096
	KI06	90 (25.1%)	68 (22.3%)	22 (41.5%)	8.858	.003
	SP06	95 (26.5%)	68 (22.3%)	27 (50.9%)	19.010	.000
	Other	93 (26.0%)	79 (25.9%)	14 (26.4%)	.006	.937

KMGP: Korean medical general practitioner, KMNPS Korean medical neuropsychiatry specialist.

**Table 8:** The herbal medicine decoction used for severe insomnia as a first choice and the difference between KMGP and KMNPS.

Category	Total (*n* = 358)	KMGP (*n* = 305)	KMNPS (*n* = 53)	*χ* ^2^	*p*
Guipi Decoction	176 (49.2%)	150 (49.2%)	26 (49.1%)	.000	.987
Renshu powder	26 (7.3%)	21 (6.9%)	5 (9.4%)	.436	.509
Tianwang Buxin pill	69 (19.3%)	48 (15.7%)	21 (39.6%)	16.556	.000
Wendan decoction	176 (49.2%)	148 (48.5%)	28 (52.8%)	.335	.563
Qingxin Lianzi decoction	60 (16.8%)	48 (15.7%)	12 (22.6%)	1.543	.214
Guizhi Jia Longgu Muli decoction	53 (14.8%)	45 (14.8%)	8 (15.1%)	.004	.949
Yokgansan	52 (14.5%)	35 (11.5%)	17 (32.1%)	15.434	.000
Si-wu Anshen decoction	35 (9.8%)	26 (8.5%)	9 (17.0%)	3.661	.056
Buxue Anshen decoction	13 (3.6%)	7 (2.3%)	6 (11.3%)	10.511	.001
Yangxin decoction	19 (5.3%)	16 (5.2%)	3 (5.7%)	.015	.901
Qutan decoction	4 (1.1%)	3 (1.0%)	1 (1.9%)	.333	.564
Suanzaoren decoction	97 (27.1%)	79 (25.9%)	18 (34.0%)	1.485	.223
Ganmaidazao decoction	32 (8.9%)	23 (7.5%)	9 (17.0%)	4.944	.026
Chaihu Jia Longgu Muli decoction	91 (25.4%)	65 (21.3%)	26 (49.1%)	18.335	.000
Xiaoyao powder	99 (27.7%)	83 (27.2%)	16 (30.2%)	.200	.655
Hwanglianjiedu decoction	43 (12.0%)	31 (10.2%)	12 (22.6%)	6.652	.010
Xiangsha Yangwei decoction	10 (2.8%)	8 (2.6%)	2 (3.8%)	.391	.822
Xiangfuzi Ba-wu decoction	14 (3.9%)	12 (3.9%)	2 (3.8%)	.003	.956
Er ShenJiao Ji Dan	1 (.3%)	1 (.3%)	0 (0.0%)	.174	.676
Qingxin Daotan decoction	9 (2.5%)	8 (2.6%)	1 (1.9%)	.100	.752
Not taking	19 (5.3%)	18 (5.9%)	1 (1.9%)	1.448	.229
Other	47 (13.1%)	40 (13.1%)	7 (13.2%)	.000	.985

KMGP: Korean medical general practitioner, KMNPS: Korean medical neuropsychiatry specialist.

**Table 9:** The herbal medicine decoction used for chronic insomnia and the difference between KMGP and KMNPS.

Category	Total (*n* = 358)	KMGP (*n* = 305)	KMNPS (*n* = 53)	*χ* ^2^	*p*
Guipi decoction	246 (68.7%)	202 (66.2%)	44 (83.0%)	5.921	.015
Renshu powder	36 (10.1%)	33 (10.8%)	3 (5.7%)	1.329	.249
Tianwang Buxin pill	65 (18.2%)	51 (16.7%)	14 (26.4%)	2.855	.091
Wendan decoction	160 (44.7%)	132 (43.3%)	28 (52.8%)	1.667	.197
Qingxin Lianzi decoction	91 (25.4%)	72 (23.6%)	19 (35.8%)	3.570	.059
Guizhi Jia Longgu Muli decoction	47 (13.1%)	39 (12.8%)	8 (15.1%)	.211	.646
Yokgansan	23 (6.4%)	17 (5.6%)	6 (11.3%)	2.481	.115
Si-wu Anshen decoction	81 (22.6%)	64 (21.0%)	17 (32.1%)	3.173	.075
Buxue Anshen decoction	64 (17.9%)	46 (15.1%)	18 (34.0%)	10.963	.001
Yangxin decoction	34 (9.5%)	28 (9.2%)	6 (11.3%)	.241	.624
Qutan decoction	5 (1.4%)	5 (1.6%)	0 (0.0%)	1.060	.588
Suanzaoren decoction	87 (24.3%)	75 (24.6%)	12 (22.6%)	.093	.760
Ganmaidazao decoction	35 (9.8%)	31 (10.2%)	4 (7.5%)	.351	.554
Chaihu Jia Longgu Muli decoction	62 (17.3%)	45 (14.8%)	17 (32.1%)	9.461	.002
Xiaoyao powder	83 (23.2%)	67 (22.0%)	16 (30.2%)	1.714	.191
Hwanglianjiedu decoction	11 (3.1%)	9 (3.0%)	2 (3.8%)	.103	.749
Xiangsha Yangwei decoction	28 (7.8%)	24 (7.9%)	4 (7.5%)	.006	.936
Xiangfuzi Ba-wu decoction	38 (10.6%)	30 (9.8%)	8 (15.1%)	1.316	.251
Er ShenJiao Ji Dan	1 (.3%)	0 (0.0%)	1 (1.9%)	5.771	.016
Qingxin Daotan decoction	12 (3.4%)	8 (2.6%)	4 (7.5%)	3.380	.066
Not taking	9 (2.5%)	8 (2.6%)	1 (1.9%)	.100	.752
Other	52 (14.5%)	46 (15.1%)	6 (11.3%)	.515	.473

KMGP: Korean medical general practitioner, KMNPS: Korean medical neuropsychiatry specialist.

**Table 10:** The assessment of treatment and the difference between KMGP and KMNPS.

Assessment methods	Total (*n* = 358)	KMGP (*n* = 305)	KMNPS (*n* = 53)	*χ* ^2^	*p*
A patient's statement	352 (98.3%)	300 (98.4%)	52 (98.1%)	.017	.897
Questionnaire	22 (6.1%)	4 (1.3%)	18 (34.0%)	83.461	.000
Polysomnography	0 (0.0%)	0 (0.0%)	0 (0.0%)	-	-
Actigraphy, etc.	0 (0.0%)	0 (0.0%)	0 (0.0%)	-	-
EEG including neurofeedback	3 (.8%)	2 (.7%)	1 (1.9%)	.824	.364
Wearable device	0 (0.0%)	0 (0.0%)	0 (0.0%)	-	-
Sleep application	2 (.6%)	2 (.7%)	0 (0.0%)	.349	.554
Dose of sleeping pill's	114 (31.8%)	86 (28.2%)	28 (52.8%)	12.625	.000
HRV	18 (5.0%)	6 (2.0%)	12 (22.6%)	40.417	.000
Change in pulse	76 (21.2%)	65 (21.3%)	11 (20.8%)	.008	.927
Change of face color	74 (20.7%)	59 (19.3%)	15 (28.3%)	2.210	.137
Other	8 (2.2%)	8 (2.6%)	0 (0.0%)	1.422	.233

KMGP: Korean medical general practitioner, KMNPS: Korean medical neuropsychiatry specialist.

**Table 11:** The difficulties in treating insomnia by Korean medicine.

Category	Total (*n* = 358)	KMGP (*n* = 305)	KMNPS (*n* = 53)	*χ* ^2^	*p*
Delayed effectiveness	218 (61.1%)	175 (57.6%)	43 (81.1%)	10.583	.005
Differential diagnosing from other mental disorder	49 (13.7%)	42 (13.8%)	7 (13.2%)	.012	.912
Objective evaluation	163 (45.5%)	142 (46.6%)	21 (39.6%)	.876	.349
Pattern identification	14 (3.9%)	14 (4.6%)	0 (0.0%)	2.532	.112
Cost burden of patients	229 (64.0%)	189 (62.0%)	40 (75.5%)	3.573	.059
Patients' negative character	21 (5.9%)	20 (6.6%)	1 (1.9%)	1.784	.182
Other	15 (4.2%)	13 (4.3%)	2 (3.8%)	.027	.870

KMGP: Korean medical general practitioner, KMNPS: Korean medical neuropsychiatry specialist.

**Table 12:** Do they have sleep hygiene education and counselling for insomnia?

Sleep hygiene education and counselling	Total (*n* = 358)	KMGP (*n* = 305)	KMNPS (*n* = 53)	*χ* ^2^	*p*
Done	176 (49.6%)	128 (42.4%)	48 (90.6%)	41.871	.000
Not done	179 (50.4%)	174 (57.6%)	5 (9.4%)		

KMGP: Korean medical general practitioner, KMNPS: Korean medical neuropsychiatry specialist.

**Table 13:** The suitable duration and cost for sleep hygiene education and counselling for insomnia.

Category	Total (*n* = 358)	KMGP (*n* = 305)	KMNPS (*n* = 53)	*t*	*p*
Minutes per session	18.02 (12.84)	17.73 (13.58)	19.53 (7.86)	−.932	.352
Number per week	1.43 (1.20)	1.46 (1.29)	1.25 (0.52)	1.167	.244
Total weeks	8.33 (4.27)	8.54 (4.37)	7.26 (3.54)	1.994	.047
Cost (KRW) per session	16126.69 (14979.97)	16182.16 (16051.49)	15815.79 (6311.10)	.139	.890

KMGP Korean medical general practitioner, KMNPS Korean medical neuropsychiatry specialist.

## Supplementary Materials

Supplementary Materials Both English and Korean versions of questionnaire contents.
