# Supplementary material for: The Pilot Survey of the Perception on the Practice Pattern, Diagnosis, and Treatment on Korean Medicine Insomnia: Focusing on the Difference between Korean Medical Neuropsychiatry Specialists and Korean Medical General Practitioners
Source: Evid Based Complement Alternat Med. 2018 Feb 11;2018:9152705. doi: 10.1155/2018/9152705 (PMC5828562; doi:10.1155/2018/9152705)
Supplement: Supplementary Materials — Both English and Korean versions of questionnaire contents. [file 9152705.f1.docx]

**Supplement 1 English Translated Version**

**Section A. General characteristic**

1. What is your age?

① Under 30 ② 30~39 ③ 40~49 ④ 50~59 ⑤ 60s or more

2. What is your sex?

① Male ② Female

3. Where is your current work place?

① KM primary clinic ② KM hospital ③ University, laboratory

④ Public health service, military service ⑤ Convalescent hospital

⑥ Leave of absence for job ⑦ other:

4. How many years have you worked as KMDs?

① Under 5 years ② 5 ~ 10 years ③ 10 ~ 20 years ④ 20 ~ 30 years ⑤ 30 years or more

B. Current Status of KMDs’ Clinical Practice on Insomnia

1. How many new patients who have insomnia as a main symptom visit your clinic a month?

① None ② Under 10 ③ 10 ~ under 30 ④ 30 ~ under 60 ⑤ 60 or more ⑥ Others:

2. How many new patients who have insomnia as an accompanying symptom visit your clinic a month?

① None ② Under 10 ③ 10 ~ under 30 ④ 30 ~ under 60 ⑤ 60 or more ⑥ Others:

3. What is your main method to diagnosis or evaluate insomnia (allow multiple response)?

① Patient’s main complaints ② Questionnaries related to insomnia PSQI or ISI)

③ EEG including neurofeedback ④ HRV

⑤ Refer to other department (polysomnography) ⑥ pulse diagnosis ⑦ Other :

4. Do you ask patients whether they take sleeping pills (anxiolytics or antidepressants) or not while you treat insomnia patients?

① Yes ② No

5. What percent of the new insomnia outpatients are taking sleeping pills (anxiolytics or antidepressants)?

① None ② Under 10% ③ 10% ~ under 30% ④ 30% ~ under 60%

⑤ 60% ~ under 90% ⑥ 90% or more

6. What percent of the insomnia outpatients have private insurance?

① None ② Under 10% ③ 10% ~ under 30% ④ 30% ~ under 60%

⑤ 60% ~ under 90% ⑥ 90% or more ⑦ Don’t know

7. Why do you think the insomnia patients want to see KMDs based on the statement of patients or your experience (allow multiple response)?

① To increase sleeping time

② To improve quality of sleep

③ To reduce or quit takin sleeping pills (anxiolytics or antidepressants)

④ To be unsatisfied with sleeping pills (anxiolytics or antidepressants)

⑤ To alleviate the related physical symptoms (headache, fatigue, dyspepsia, neck pain, shoulder pain, back pain)

⑥ Other :

8. This is the table shows the frequently used code(Korean standard classidication of disease, KCD) for insomnia disorder. Please list in your order of frequency of use from 1st to 3rd. You may write other codes besides codes in the table below.

| KCD | record | KCD | record |
| --- | --- | --- | --- |
| G470 Disorders of initiating and maintaining sleep(insomnia) |  | F510 Nonorganic sleep disorders |  |
| G472 Disorder of the sleep-wake schedule |  | F512 Nonorganic disorder of the sleep-wake schedule |  |
| G478 Other sleep disorder |  | F518 Nonorganic disorder of the sleep-wake schedule |  |
| G479 Sleep disorder, unspecified |  | F519 Nonorganic sleep disorder, unspecified |  |

9. What kind of KM diagnostic method (pattern identification) do you usually choose when you treat insomnia patients?

① Visceral pattern identification ② Meridian and collateral pattern identification

③ Eight principles pattern identification ④ Six-Meridian Pattern Identification

⑤ Defense, Qi, Nutrient and Blood Pattern Identification

⑥ Constitutional pattern identification ⑦ Other

10. Please select all of the treatments for insomnia you use among the following example (allow multiple response).

① Acupunture ② Electro-acupuncture ③ Auricular acupuncture ④ Intradermal Acupuncture ⑤ Warm needling acupuncture ⑥ Fire needling acupuncture ⑦ Miniscalpel acupuncture

⑧ Pharmacopuncture(except Bee Venom Pharmacopuncture) ⑨ Bee Venom Pharmacopuncture

⑩ Chuna Manual Therapy (practice by the doctor) ⑪ Dao-Yin exercise(self-practice by the doctor)

⑫ insured Herbal Extract granules ⑬ Uninsured Herbal Extract granules ⑭ Herbal medicine

⑮ Wet cupping ⑯ Dry cupping ⑰ Moxibustion ⑱ Needle-embedding Therapy ⑲ Ice&Hot pack

⑳ Meditation ㉑ TENS ㉒ ICT ㉓ Deep heat- diathermy ㉔ Laser ㉕ Aromatherapy

㉖ Psychotherapy ㉗ Biofeedback

Other:

11. Please record the proportion of TCM treatments for insomnia, classified as the following criteria (total=100%).

| Acupunture | Herbal medicine | Insured Herbal Extract granules | Uninsured Herbal Extract granules | Moxibustion | Cupping | Other | Total |
| --- | --- | --- | --- | --- | --- | --- | --- |
| % | % | % | % | % | % | % | =100% |

12. What are the main treatment methods of acupuncture and acuppoints used for treatment of insomnia (allow multiple response)?

| Acupunture | Treatment methods | ①General acupuncture ②Auricular acupuncture  ③Head-acupuncture ④O-Haeng acupuncture  ⑤Constitutional acupuncture ⑥Dong-Si acupuncture  ⑦Sa-am acupuncture ( ) ⑨Other |
| --- | --- | --- |
|  | Acuppoints | ⓐ GV20 ⓑ Sishencong ⓒ HT07 ⓓ HT08 ⓔ PC06 ⓕ ST36 ⓖ KI06 ⓗ BL62 ⓘ SP06 ⓙ Other |
| Not use acupunture | Reason |  |

13. Please select all of herbal medicine use for acute insomnia (<1 wks).

① Guipi Decoction ② Renshu powder ③ Tianwang Buxin pill ④ Wendan decoction

⑤ Qingxin Lianzi decoction ⑥ Guizhi Jia Longgu Muli decoction ⑦ Yokgansan

⑧ Si-wu Anshen decoction ⑨ Buxue Anshen decoction ⑩ Yangxin decotion ⑪ Qutan decoction

⑫ Suanzaoren decoction ⑬ Ganmaidazao decoction ⑭ Chaihu Jia Longgu Muli decoction

⑮ Xiaoyao powder ⑯ Hwanglianjiedu decoction ⑰ Xiangsha Yangwei decoction

⑱ Xiangfuzi Ba-wu decoction ⑲ Er ShenJiao Ji Dan ⑳ Qingxin Daotan decoction ㉑ Not taking

㉒ Other

14. Please select all of herbal medicine use for non-acute insomnia (>1 wks)

① Guipi Decoction ② Renshu powder ③ Tianwang Buxin pill ④ Wendan decoction

⑤ Qingxin Lianzi decoction ⑥ Guizhi Jia Longgu Muli decoction ⑦ Yokgansan

⑧ Si-wu Anshen decoction ⑨ Buxue Anshen decoction ⑩ Yangxin decotion ⑪ Qutan decoction

⑫ Suanzaoren decoction ⑬ Ganmaidazao decoction ⑭ Chaihu Jia Longgu Muli decoction

⑮ Xiaoyao powder ⑯ Hwanglianjiedu decoction ⑰ Xiangsha Yangwei decoction

⑱ Xiangfuzi Ba-wu decoction ⑲ Er ShenJiao Ji Dan ⑳ Qingxin Daotan decoction ㉑ Not taking

㉒ Other

15. What is method of the assessment of treatment (allow multiple responses)?

① Patient’s statement

② Questionnaire(PSQI, ISI) ③ Polysonography ④ Actigraphy ⑤ EEG ⑥ Wearable device

⑦ Sleep application(in smart phone) ⑧ Dose of sleeping pill's ⑨ HRV ⑩ Change in pulse

⑪ Change of face color ⑫ Other:

16. How long does it take to appear treatment response based on your clinical experience?

① Less than 1 week ② Less than 1-2 weeks ③ Less than 2-3 weeks ④ Less than 3-4 weeks

⑤ More than 4 weeks ⑥ Other

17. How long does minimal duration of treatment on your clinical experience?

① Less than 1 week ② Less than 1-4 weeks ③ Less than 4-8 weeks ④ Less than 8-12 weeks

⑤ Less than 12-24 weeks ⑥ More than 24 weeks ⑦ Other

18. What are the difficulties in medical treatment for insomnia (allow multiple responses)?

① Delayed effectiveness ② Differential diagnosing from other mental disorder

③ Difficulty in objective evaluation ④ Difficulty in Pattern identification

⑤ Cost burden of patients ⑥ Patients’ negative character

⑦ Other

19. What was the most important factor in case appears good treatment response (allow multiple responses)?

① Rapport with patient

② Take steady herbal medicine

③ Continuous acupuncture treatment

④ Changes in patient's external environment(Stressor disappears)

⑤ Sleeping hygiene education and counseling

⑥ Other

20. Do you use sleeping hygiene education and counseling to manage patients?

① Yes ② No

21. What is reasonable time and cost based on your clinical experience if you do sleeping hygiene education and counseling in the future?

| Time | Number of times | Total duration | Cost |
| --- | --- | --- | --- |
| min/1 session | session/1 week | week | won/1 session |

**Supplement 2 Korean Version**

A. 일반적 사항

1. 귀하의 연령은?

① 30대 이하 ② 30대 ③ 40대 ④ 50대 ⑤ 60대 이상

2. 귀하의 성별은?

① 남 ② 여

3. 귀하의 소속기관은?

① 한의원 ② 한방병원 ③ 대학, 연구소

④ 보건복지부(공보의), 국방부(군의관) ⑤ 요양병원

⑥ 휴직 중 ⑦기타:

4. 귀하의 임상경험은 몇 년입니까?

① 5년 미만 ② 5년 ~ 10년 미만 ③ 10년 ~ 20년 미만 ④ 20년 ~ 30년 미만

⑤ 30년 이상

B. 불면장애의 한의진료현황

1. 불면증(장애)을 주증(主症)으로 내원하는 환자의 수는 1개월에 몇 명 정도(초진 기준) 입니까?

① 없음 ② 10명 이하 ③ 10명 이상 ~ 30명 미만 ④ 30명 이상 ~ 60명 미만

⑤ 60명 이상 ⑥기타:

2. 불면증(장애)을 차증(次症) 혹은 동반 증상으로 호소하는 환자의 수는 1개월에 몇 명 정도(초진 기준) 입니까?

① 없음 ② 10명 이하 ③ 10명 이상 ~ 30명 미만 ④ 30명 이상 ~ 60명 미만

⑤ 60명 이상 ⑥기타:

3. 불면증(장애) 환자 진단 혹은 평가도구로 무엇을 사용하십니까?(복수 응답 가능)

① 환자의 주호소 ② 불면 관련 설문지(PSQI 혹은 ISI 등)

③ 뇌파를 활용한 도구(뉴로피드백 등) ④ HRV (수양명경락기능검사 등)

⑤ 타과 검사 의뢰 (polysomnography 등) ⑥ 맥증(맥전도 등)

⑦ 기타 :

4. 불면증(장애) 진료시 수면제(항불안제, 항우울제 등 포함) 복용 여부를 확인하십니까?

① 예 ② 아니오

5. 불면을 주소로 내원한 환자 중 수면제(항불안제, 항우울제 등 포함)를 복용 중인 환자 비율(초진환자 기준)은 어느 정도입니까?

① 없음 ② 10% 미만 ③ 10% ~ 30% 미만 ④ 30% 이상 ~ 60% 미만

⑤ 60% ~ 90% 미만 ⑥ 90% 이상

6. 불면을 주소로 외래 내원한 환자 중 사보험(실비, 보장성 보험 등)으로 혜택을 받는 비율은 어느 정도입니까?

① 없음 ② 10% 미만 ③ 10% ~ 30% 미만 ④ 30% 이상 ~ 60% 미만

⑤ 60% ~ 90% 미만 ⑥ 90% 이상 ⑦ 모르겠다

7. 환자의 진술 또는 진료경험에 의거하여, 불면증 환자가 한의치료를 원하는 이유는 무엇입니까?(복수 응답 가능)

① 수면 시간 증가를 희망

② 수면 질의 개선을 희망

③ 수면제(항우울제, 항불안제 등)의 감량 혹은 중단을 위해

④ 수면제(항우울제, 항불안제 등)의 효과가 불충분하다고 느껴

⑤ 불면에 동반된 기타 신체증상(두통, 피로, 소화장애, 항강통, 견배통 등)을 완화하기 위해

⑥ 기타 :

8. 아래 표는 불면 환자에 빈용되는 상병코드(한국표준질병사인분류)입니다. 귀하의 불면증주된 사용 코드를 1,2,3 수위로 써 주십시오. 제시된 상병 이외의 것을 적어도 무방합니다.

| 주상병 코드 | 표시 | 주상병 코드 | 표시 |
| --- | --- | --- | --- |
| G470 수면개시 및 유지장애(불면증) |  | F510 비기질성 불면증 |  |
| G472 수면-각성 양상의 장애 |  | F512수면-각성 주기의 비기질성 장애 |  |
| G478 기타 수면장애 |  | F518 기타 비기질성 수면장애 |  |
| G479 상세불명의 수면장애 |  | F519 상세불명의 비기질성 수면장애 |  |
| 기타 |  | 기타 |  |
| 기타 |  | 기타 |  |

9. 불면증(장애) 치료시 주로 어떤 변증(한의진단) 방법을 사용합니까?

① 장부변증 ② 경락변증 ③ 팔강변증 ④ 육경변증 ⑤ 위기영혈변증

⑥ 체질을 이용한 진단 ⑦ 기타:

10. 불면증(장애) 환자에게 사용하는 한의치료방법을 모두 선택해 주십시오.

① 침 ② 전침 ③ 이침 ④ 피내침 ⑤ 온침 ⑥ 화침 ⑦ 도침 ⑧ 약침(봉약침제외) ⑨ 봉약침 ⑩ 추나(한의사가 시행) ⑪ 도인(환자가 수행) ⑫ 56종의 보험한약제제 ⑬ 비보험 한약제제 ⑭ 한약(탕약) ⑮ 습식부항 ⑯ 건식부항 ⑰ 뜸치료 ⑱ 매선 ⑲ Ice&Hot pack ⑳명상

㉑ TENS ㉒ ICT ㉓ 초음파, 초단파 등 심부투열기 ㉔ 레이저 치료 ㉕ 향기치료

㉖ 심리(상담) 치료 ㉗ 생기능자기조절훈련(뉴로피드백 등 기기 이용)

기타:

11. 불면증(장애) 환자에 사용하는 한의치료방법을 아래와 같이 분류할 때, 사용 비율을 적어주십시오(총합이 100%).

| 침 | 탕약 | 보험  과립제 | 비보험  한약제재 | 뜸 | 부항 | 기타 | 총합 |
| --- | --- | --- | --- | --- | --- | --- | --- |
| % | % | % | % | % | % | % | =100% |

12. 불면증(장애)의 치료 시 주로 활용하는 침치료방법과 혈위(穴位)는?(복수응답 가능)

| 침치료 시행 | 치료법 | ①체침 ②이침 ③두침 ④오행침법 ⑤체질침법 ⑥동씨침법  ⑦사암침법( )  ⑨기타 : |
| --- | --- | --- |
|  | 혈위 | ⓐ백회 ⓑ사신총 ⓒ신문 ⓓ소부 ⓔ내관 ⓕ족삼리 ⓖ신맥 ⓗ조해 ⓘ삼음교ⓙ기타, 특효혈: |
| 침치료 미시행 | 이유 |  |

13. 귀하께서 급성기 불면증(1주일 미만, 연속 불면)에 사용하는 한약(탕약)을 모두 골라주십시오.

① (가미)귀비탕 ② 인숙산 ③ 천왕보심단 ④ (가미)온담탕 ⑤ (가감)청심연자탕

⑥ 계지가용골모려탕 ⑦ (가미)억간산 ⑧ (가미)사물안신탕 ⑨ (가미)보혈안신탕 ⑩ 양심탕

⑪거담청신탕 ⑫ 산조인탕 ⑬ 감맥대조탕 ⑭ 시호가용골모려탕 ⑮ (가미)소요산

⑯ 황련해독탕 ⑰향사양위탕 ⑱향부자팔물탕 ⑲이신교제단 ⑳청심도담탕 ㉑ 처방경험 없음

㉒ 기타

14. 귀하께서 불면증(장애) 비급성기에 사용하는 한약(탕약)을 모두 골라주십시오.

① (가미)귀비탕 ② 인숙산 ③ 천왕보심단 ④ (가미)온담탕 ⑤ (가감)청심연자탕

⑥ 계지가용골모려탕 ⑦ (가미)억간산 ⑧ (가미)사물안신탕 ⑨ (가미)보혈안신탕 ⑩ 양심탕

⑪거담청신탕 ⑫ 산조인탕 ⑬ 감맥대조탕 ⑭ 시호가용골모려탕 ⑮ (가미)소요산

⑯ 황련해독탕 ⑰향사양위탕 ⑱향부자팔물탕 ⑲이신교제단 ⑳청심도담탕 ㉑ 처방경험 없음

㉒ 기타

15. 귀하의 불면증(장애)의 치료효과를 평가하는 방법은 무엇입니까?(복수응답 가능)

① 수면시간 증가, 수면의 질 향상 등에 대한 환자의 진술

② 설문지(PSQI, ISI 등)의 변화 ③ 수면다원검사(Polysonography)

④ actigraphy 등 ⑤ 뇌파검사 ⑥ wearable device(인바디밴드, 미밴드, 핏빗 등)

⑦ 수면어플(스마트폰, iPad 등) ⑧ 수면제 복용량의 감소

⑨ HRV ⑩ 맥의 변화(맥전도 포함) ⑪ 기혈색의 변화

⑫기타 :

16. 귀하의 진료 경험에 근거하여, 호전반응(예, 수면시간 증가, 수면의 질 향상, 일상생활 기능 향상, 수면제 복용량 감소 등등)이 나타나기 까지 걸리는 시간은 어느 정도입니까?

① 1주 미만 ② 1주 ~ 2주 미만 ③ 2주 ~ 3주 미만 ④ 3주 ~ 4주 미만 ⑤ 4주 이상

⑥ 기타:

17. 귀하께서 생각하는 최소한의 불면증(장애)의 치료기간은? *

① 1주 미만 ② 1주~4주 미만 ③ 4주 ~2개월 미만 ④ 2개월 ~ 3개월 미만

⑤ 3개월~6개월 미만 ⑥ 6개월 이상 ⑦ 기타 :

18. 불면증(장애) 진료 시 어려운 점은 무엇입니까?(복수응답 가능)

① 치료의 효과가 신속하지 않음 ② 다른 정신장애와 감별 진단의 어려움

③ 객관적인 호전도 평가의 어려움 ④ 불면 변증이 어려움

⑤ 환자의 비용 부담 ⑥ 환자와의 면담(근거 부족)이 어려움

⑦기타:

19. 불면증(장애) 치료경과가 좋았던 경우, 가장 중요한 요소는 무엇이었습니까?

① 환자와의 라포

② 꾸준한 한약 복용

③ 적극적인 침구치료

④ 환자 외부 환경의 변화(스트레스 요인 제거)

⑤ 꾸준한 수면지도 상담

⑥ 기타:

20. 불면 환자 치료시 수면 개선을 위한 교육이나 지도면담(수면위생교육 등)를 시행하고 있습니까?

① 시행함 ② 시행하지 않음

21. 향후 수면교육면담(수면위생교육 등)을 시행한다면, 적정 시간과 청구비용은 어느 정도가 적당하다고 생각하십니까?

| 시간 | 횟 수 | 총 기간 | 비용 |
| --- | --- | --- | --- |
| 분/1회 | 회/1주 | 주 | 원/1회 |
